# A trinuclear Fe–Fe–Ni complex formed by ligand reshuffling

**DOI:** 10.1107/S1600536811017892

**Published:** 2011-05-20

**Authors:** Ariel Peleg, Wenfeng Lo, Jianfeng Jiang

**Affiliations:** a Department of Chemistry, Yeshiva University, 500 West 185th Street, New York, NY 10033, USA; b Department of Chemistry and Chemical Biology, Harvard University, 12 Oxford Street, Cambridge, MA 02138, USA

## Abstract

The title complex, dicarbonyl-3κ^2^ *C*-(μ_3_-3,6-dimethyl-3,6-diaza­octane-1,8-dithiol­ato-1:2:3κ^7^ *S*:*S*,*N*,*N*′,*S*′:*S*,*S*′)(μ_2_-3,6-di­methyl-3,6-diaza­octane-1,8-dithiol­ato-1:2κ^5^ *S*,*N*,*N*′,*S*′:*S*)-1,2-diiron(II)-3-nickel(0) [Fe_2_Ni(C_8_H_18_N_2_S_2_)_2_(CO)_2_], is the second example showing *M*(μ-S*R*)_2_Ni^0^(CO)_2_ coordination (*M* = any metal atom). Both Fe^II^ ions are five-coordinated in distorted trigonal–bipyramidal geometries by two N atoms and three S atoms. The Ni atom is four-coordinated in a distorted tetra­hedral geometry by two S atoms and two carbonyl ligands. One of the 3,6-dimethyl-3,6-diaza­octane-1,8-dithiol­ate ligands is disordered, the major component having a refined occupancy of 0.873 (2). The Fe⋯Fe distance is 3.0945 (3)Å and the Ni⋯Fe distance is 2.8505 (3) Å.

## Related literature

For the structure of [Fe^II^(dsdm)Ni^0^(CO)_3_]_2_ (dsdm = 3,6-dimethyl-3,6-diazaoctane-1,8-dithiolato), see: Bouwman *et al.* (1999 ▶). For the structure of [Ni^II^(N_2_S_2_′)Ni^0^(CO)_2_] (N_2_S_2_′ = 4,7-diazadecane-3,8-dione-1,10-dithiolato), see: Linck *et al.* (2003 ▶). For the structure of [Fe^II^(dsdm)]_2_, see: Hu & Lippard (1974 ▶). The synthesis of the starting materials [Et_4_N][Fe^II^(CN)_2_(CO)_3_I] and [Ni^II^(dsdm)] has been described by Jiang *et al.* (2009 ▶) and Turner *et al.* (1990 ▶). For structures of Ni–Fe hydrogenase active sites, see: Fontecilla-Camps *et al.* (2007 ▶). Structure checking was performed using *PLATON* (Spek, 2009 ▶).
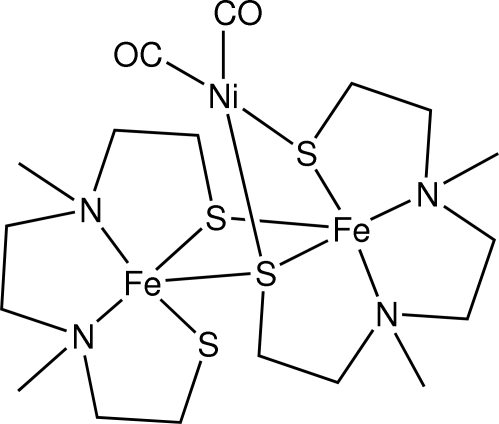


## Experimental

### 

#### Crystal data

[Fe_2_Ni(C_8_H_18_N_2_S_2_)_2_(CO)_2_]
*M* *_r_* = 639.16
Triclinic, 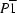

*a* = 8.4051 (3) Å
*b* = 12.7146 (5) Å
*c* = 13.3451 (5) Å
α = 70.475 (1)°
β = 83.208 (1)°
γ = 79.309 (1)°
*V* = 1318.38 (9) Å^3^
*Z* = 2
Mo *K*α radiation
μ = 2.13 mm^−1^
*T* = 173 K
0.35 × 0.10 × 0.10 mm

#### Data collection

Bruker SMART CCD area-detector diffractometer
Absorption correction: multi-scan (*SADABS*; Sheldrick, 2004 ▶) *T*_min_ = 0.774, *T*_max_ = 0.808
41649 measured reflections
6596 independent reflections
5863 reflections with *I* > 2σ(*I*)
*R*_int_ = 0.026

#### Refinement

*R*[*F* ^2^ > 2σ(*F* ^2^)] = 0.022
*wR*(*F* ^2^) = 0.056
*S* = 1.07
6596 reflections
301 parameters
2 restraints
H-atom parameters constrained
Δρ_max_ = 0.84 e Å^−3^
Δρ_min_ = −0.31 e Å^−3^

### 

Data collection: *SMART* (Bruker, 2003 ▶); cell refinement: *SAINT* (Bruker, 2003 ▶); data reduction: *SAINT*; program(s) used to solve structure: *SHELXS97* (Sheldrick, 2008 ▶); program(s) used to refine structure: *SHELXL97* (Sheldrick, 2008 ▶); molecular graphics: *ORTEP-3 for Windows* (Farrugia, 1997 ▶); software used to prepare material for publication: *publCIF* (Westrip, 2010 ▶).

## Supplementary Material

Crystal structure: contains datablocks I, global. DOI: 10.1107/S1600536811017892/pk2319sup1.cif

Structure factors: contains datablocks I. DOI: 10.1107/S1600536811017892/pk2319Isup2.hkl

Additional supplementary materials:  crystallographic information; 3D view; checkCIF report

## Figures and Tables

**Fig. 1.**
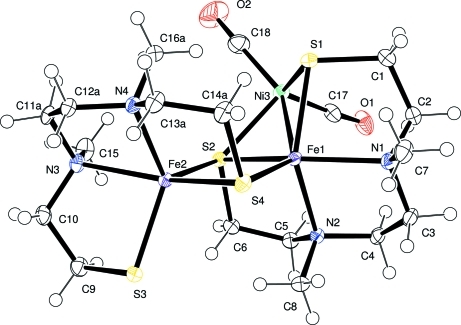
: Ellipsoid plot of [FeII(dsdm)FeII(dsdm)Ni0(CO)2] at 50% probability level. The minor disorder component is omitted for the sake of clarity.

## supplementary crystallographic information

### Comment

We studied the reaction between [Ni^II^(dsdm)] (Turner *et al.*, 1990) and (Et_4_N)[Fe^II^(CN)_2_(CO)_3_I] (Jiang *et al.*, 2009) in methanol solution in an attempt to synthesize a dithiolate bridged Ni-Fe complex [Ni^II^(dsdm)Fe^II^(CN)_2_(CO)_2_] to mimic the active site structure of Ni-Fe hydrogenase (Fontecilla-Camps *et al.*, 2007), However, an insoluble material was obtained. Solid state infrared spectroscopy of the material has shown the presence of both CO and CN ligands.  This material was then treated with LiHBEt_3_ in THF to give a black-colored solution. Surprisingly, [Fe^II^(dsdm)]_2_Ni^0^(CO)_2_] (Fig. 1) was isolated upon diffusion of diethyl ether to the black colored solution.

Reshuffling of ligands on [Ni^II^(dsdm)] is not unprecedented.  Bouwman has shown that a tetranuclear complex [Fe^II^(dsdm)Ni^0^(CO)_3_]_2_ is isolated by the reaction between [Ni^II^(dsdm)] and K[HFe^0^(CO)_4_] in refluxing ethanol (Bouwman *et al.*, 1999).  Nickel is reduced by the low oxidation iron species in solution.  In our system, a similar reshuffling occured, and nickel was reduced by hydride in THF solution.

This molecule crystallizes in the triclinic space group P-1 with one molecule per asymmetric unit.  Part of the molecule is disordered.  This disorder can be described as an approximate mirror operation about a plane along N4, N3, S4 and Fe2.  The ratio between the two components of this disorder was refined freely, and converged at 0.873 (2).  Bond lengths and angles between atoms of the major components of the disorders are determined with significantly higher accuracy than for those between the corresponding atoms of the minor components.

Nevertheless, the structure of [Fe^II^(dsdm)]_2_Ni^0^(CO)_2_] shows several interesting features. It is only the second example of a M(µ-SR)_2_Ni(CO)_2_ coordination other than (Et_4_N)_2_[Ni^II^(S_2_N_2_')Ni^0^(CO)_2_] (S_2_N_2_' = µ_2_-4,7-diazadecane-3,8-dione-1,10-dithiolato-N,N',S,S,S',S') made by Rauchfuss (Linck *et al.*, 2003).

The trinuclear Fe-Fe-Ni complex is not symmetric. The Ni(CO)_2_ unit is bridged by two sulfur atoms from one [Fe^II^(dsdm)] unit.  One such sulfur atom forms an additional bond to the adjacent iron.  All four sulfur atoms in this molecule are different in their metal-coordination nature.  S3 only coordinates to Fe2; S4 bridges between Fe1 and Fe2; S1 bridges between Fe1 and Ni3; S2 bridges among Fe1, Fe2 and Ni3. Even though both [Fe^II^(dsdm)] units are distorted trigonal bipyramidal, their geometries are significantly different. The structure of the non-Ni bridging [Fe(dsdm)] unit is similar to the tetramer, [Fe^II^(dsdm)Ni^0^(CO)_3_]_2_, reported (Bouwman *et al.*, 1999) and the parent [Fe^II^(dsdm)]_2_ dimer reported by Lippard (Hu and Lippard, 1974). In the non-Ni-bridging [Fe^II^(dsdm)] unit, the axial bonds are much longer than the equatorial bonds, *i.e.*, Fe2-N3 (2.381 (2)Å) > Fe2-N4 (2.160 (2)Å), and Fe2-S4 (2.4511 (4)Å) > Fe2-S3 (2.3122 (4)Å) and Fe2-S2 (2.3919 (4)Å).  However, the Ni-bridging [Fe(dsdm)] shows more distorted trigonal bipyramidal geometry.  Significantly, the difference between the axial Fe1-N1 (2.241 (2)Å) and the equatorial Fe1-N2 (2.204 (2)Å) is not as substantial.

The Fe-Fe distance is 3.0945 (3)Å, which is similar to that in the tetranuclear Fe^II^(dsdm)Ni^0^(CO)_3_]_2_ and shorter than that in the [Fe^II^(dsdm)]_2_ dimer.  The Ni-Fe distance is 2.8505 (3)Å, which is similar to the Ni-Ni distance in (Et_4_N)_2_[Ni(S_2_N_2_')Ni(CO)_2_].

### Experimental

Anhydrous methanol, THF, diethyl ether and LiHBEt_3_ (1*M* solution in THF) was purchased from Acros.  [Ni^II^(dsdm)] and (Et_4_N)[Fe^II^(CN)_2_(CO)_3_I] were prepared according to published procedure (Turner *et al.*, 1990, Jiang *et al.*, 2009)

To 0.265 g (1.0 mmol) [Ni^II^(dsdm)] dissolved in 10 ml methanol, a solution of 0.449 g (1.00 mmol) [Et_4_N][Fe^II^(CN)_2_(CO)_3_I] in 5 ml methanol was added.  The reaction mixture was kept stirring for 2 h and a brown-colored precipitate formed. The precipitate was collected by filtration, washed with 5 ml methanol 3 times and dried under vacuum to afford 0.300 g light-brown colored powder (IR (ATR): 1983, 2036, 2110 cm^-1^).  The identity of this brown powder has not been determined. 0.400 ml LiHBEt_3_ (1*M* in THF) was added to 0.086 g of this brown powder suspended in 2 ml THF, and the powders dissolved and the color turned black. The mixture was kept stirring for 2 h, and 6 ml diethyl ether was carefully added. The product, [Fe^II^(dsdm)]_2_Ni^0^(CO)_2_], (0.029 g) was isolated as black needles after 2 days. (IR (THF): 1873, 1906 cm^-1^)

### Refinement

All hydrogen atoms were included at geometrically calculated positions and refined using a riding model.  Isotropic displacement parameters of hydrogen atoms were fixed to 1.2 times the U_eq_ value of the atoms they are linked to (1.5U_eq_ for methyl groups).  The final structure was checked for missing symmetry using PLATON (Spek, 2009).

Disorder of the molecule was refined with the help of 2 restraints on the bond distances N3-C11B and S4-C14B.  In addition, all minor-component atoms were constrained to have identical anisotropic displacement parameters to their respective major-component counterparts.  All non-hydrogen atoms were refined anisotropically.

### Crystal data

**Table:**

[Fe_2_Ni(C_8_H_18_N_2_S_2_)_2_(CO)_2_]	*Z* = 2
*M**_r_* = 639.16	*F*(000) = 664
Triclinic, *P*1	*D*_x_ = 1.610 Mg m^−^^3^
Hall symbol: -P 1	Mo *K*α radiation, λ = 0.71073 Å
*a* = 8.4051 (3) Å	Cell parameters from 300 reflections
*b* = 12.7146 (5) Å	θ = 2.5–28.4°
*c* = 13.3451 (5) Å	µ = 2.13 mm^−^^1^
α = 70.475 (1)°	*T* = 173 K
β = 83.208 (1)°	Needle, black
γ = 79.309 (1)°	0.35 × 0.10 × 0.10 mm
*V* = 1318.38 (9) Å^3^	

### Data collection

**Table:**

Bruker SMART CCD area-detector diffractometer	6596 independent reflections
Radiation source: fine-focus sealed tube	5863 reflections with *I* > 2σ(*I*)
graphite	*R*_int_ = 0.026
φ and ω scans	θ_max_ = 28.4°, θ_min_ = 1.6°
Absorption correction: multi-scan (*SADABS*; Sheldrick, 2004)	*h* = −11→11
*T*_min_ = 0.774, *T*_max_ = 0.808	*k* = −16→16
41649 measured reflections	*l* = −17→17

### Refinement

**Table:**

Refinement on *F*^2^	Primary atom site location: structure-invariant direct methods
Least-squares matrix: full	Secondary atom site location: difference Fourier map
*R*[*F*^2^ > 2σ(*F*^2^)] = 0.022	Hydrogen site location: inferred from neighbouring sites
*wR*(*F*^2^) = 0.056	H-atom parameters constrained
*S* = 1.07	*w* = 1/[σ^2^(*F*_o_^2^) + (0.0257*P*)^2^ + 0.7211*P*] where *P* = (*F*_o_^2^ + 2*F*_c_^2^)/3
6596 reflections	(Δ/σ)_max_ = 0.001
301 parameters	Δρ_max_ = 0.84 e Å^−^^3^
2 restraints	Δρ_min_ = −0.31 e Å^−^^3^

### Special details

**Table:**

Geometry. All esds (except the esd in the dihedral angle between two l.s. planes) are estimated using the full covariance matrix. The cell esds are taken into account individually in the estimation of esds in distances, angles and torsion angles; correlations between esds in cell parameters are only used when they are defined by crystal symmetry. An approximate (isotropic) treatment of cell esds is used for estimating esds involving l.s. planes.
Refinement. Refinement of F^2^ against ALL reflections. The weighted R-factor wR and goodness of fit S are based on F^2^, conventional R-factors R are based on F, with F set to zero for negative F^2^. The threshold expression of F^2^ > 2σ(F^2^) is used only for calculating R-factors(gt) etc. and is not relevant to the choice of reflections for refinement. R-factors based on F^2^ are statistically about twice as large as those based on F, and R- factors based on ALL data will be even larger.

### Fractional atomic coordinates and isotropic or equivalent isotropic displacement parameters (Å^2^)

**Table:**

	*x*	*y*	*z*	*U*_iso_*/*U*_eq_	Occ. (<1)
Fe1	0.16309 (2)	0.133392 (18)	0.257975 (16)	0.01240 (5)	
Fe2	0.09584 (2)	0.348721 (18)	0.325379 (16)	0.01218 (5)	
Ni3	0.28724 (2)	0.242434 (17)	0.047760 (16)	0.01672 (5)	
S1	0.43299 (4)	0.11355 (3)	0.18653 (3)	0.01652 (8)	
S2	0.10001 (4)	0.33301 (3)	0.15138 (3)	0.01364 (7)	
S3	−0.16068 (4)	0.39578 (3)	0.39741 (3)	0.01589 (8)	
S4	0.14979 (5)	0.14843 (3)	0.43141 (3)	0.01533 (8)	
C1	0.4545 (2)	−0.02863 (14)	0.17643 (14)	0.0227 (3)	
H1A	0.5104	−0.0291	0.1087	0.027*	
H1B	0.5207	−0.0810	0.2325	0.027*	
C2	0.29216 (19)	−0.06906 (14)	0.18532 (13)	0.0200 (3)	
H2A	0.2377	−0.0295	0.1199	0.024*	
H2B	0.3114	−0.1490	0.1936	0.024*	
C3	0.02134 (19)	−0.07559 (13)	0.27173 (13)	0.0176 (3)	
H3A	−0.0332	−0.0923	0.3422	0.021*	
H3B	0.0314	−0.1417	0.2492	0.021*	
C4	−0.07916 (19)	0.02363 (13)	0.19473 (13)	0.0170 (3)	
H4A	−0.0334	0.0330	0.1226	0.020*	
H4B	−0.1889	0.0082	0.1987	0.020*	
C5	−0.14768 (18)	0.22706 (13)	0.12884 (12)	0.0158 (3)	
H5A	−0.2637	0.2297	0.1283	0.019*	
H5B	−0.0978	0.2168	0.0627	0.019*	
C6	−0.11543 (18)	0.33834 (13)	0.13483 (12)	0.0158 (3)	
H6A	−0.1801	0.3552	0.1944	0.019*	
H6B	−0.1478	0.3985	0.0702	0.019*	
C7	0.2542 (2)	−0.12185 (14)	0.37795 (14)	0.0229 (3)	
H7A	0.1806	−0.1121	0.4360	0.034*	
H7B	0.3559	−0.1004	0.3830	0.034*	
H7C	0.2713	−0.1996	0.3810	0.034*	
C8	−0.18756 (18)	0.12685 (14)	0.31805 (12)	0.0168 (3)	
H8A	−0.2950	0.1178	0.3084	0.025*	
H8B	−0.1918	0.1964	0.3326	0.025*	
H8C	−0.1433	0.0648	0.3767	0.025*	
C9	−0.1930 (2)	0.54613 (14)	0.32196 (14)	0.0213 (3)	
H9A	−0.2153	0.5564	0.2493	0.026*	
H9B	−0.2860	0.5840	0.3536	0.026*	
C10	−0.04347 (19)	0.59781 (13)	0.32169 (13)	0.0193 (3)	
H10A	−0.0647	0.6784	0.2848	0.023*	
H10B	−0.0214	0.5869	0.3945	0.023*	
C15	0.0975 (2)	0.59951 (14)	0.15256 (13)	0.0237 (3)	
H15A	0.1044	0.6783	0.1340	0.036*	
H15B	−0.0019	0.5911	0.1293	0.036*	
H15C	0.1878	0.5634	0.1185	0.036*	
C17	0.2083 (2)	0.18224 (14)	−0.03214 (13)	0.0216 (3)	
C18	0.4093 (2)	0.34856 (16)	−0.02072 (14)	0.0274 (4)	
C11A	0.2515 (4)	0.5652 (2)	0.3033 (3)	0.0152 (6)	0.873 (2)
H11A	0.2306	0.6334	0.3232	0.018*	0.873 (2)
H11B	0.3338	0.5747	0.2449	0.018*	0.873 (2)
C12A	0.3122 (2)	0.46365 (15)	0.39850 (14)	0.0166 (3)	0.873 (2)
H12A	0.4172	0.4723	0.4153	0.020*	0.873 (2)
H12B	0.2376	0.4622	0.4601	0.020*	0.873 (2)
C13A	0.3720 (2)	0.26051 (15)	0.47315 (14)	0.0187 (4)	0.873 (2)
H13A	0.3039	0.2722	0.5336	0.022*	0.873 (2)
H13B	0.4835	0.2593	0.4864	0.022*	0.873 (2)
C14A	0.3555 (3)	0.1464 (4)	0.4646 (2)	0.0190 (7)	0.873 (2)
H14A	0.3792	0.0876	0.5318	0.023*	0.873 (2)
H14B	0.4329	0.1296	0.4100	0.023*	0.873 (2)
C16A	0.4504 (2)	0.35148 (16)	0.28764 (14)	0.0179 (4)	0.873 (2)
H16A	0.4154	0.4095	0.2234	0.027*	0.873 (2)
H16B	0.4629	0.2790	0.2776	0.027*	0.873 (2)
H16C	0.5523	0.3632	0.3045	0.027*	0.873 (2)
C11B	0.258 (3)	0.544 (2)	0.317 (3)	0.0152 (6)	0.127 (2)
H11C	0.2967	0.6154	0.2801	0.018*	0.127 (2)
H11D	0.2327	0.5404	0.3907	0.018*	0.127 (2)
C12B	0.3875 (15)	0.4560 (10)	0.3141 (10)	0.0166 (3)	0.127 (2)
H12C	0.4824	0.4642	0.3440	0.020*	0.127 (2)
H12D	0.4161	0.4556	0.2416	0.020*	0.127 (2)
C13B	0.4403 (15)	0.2509 (10)	0.3774 (10)	0.0187 (4)	0.127 (2)
H13C	0.5410	0.2506	0.4064	0.022*	0.127 (2)
H13D	0.4649	0.2498	0.3049	0.022*	0.127 (2)
C14B	0.368 (2)	0.145 (4)	0.444 (3)	0.0190 (7)	0.127 (2)
H14C	0.3846	0.1305	0.5184	0.023*	0.127 (2)
H14D	0.4285	0.0809	0.4243	0.023*	0.127 (2)
C16B	0.3161 (15)	0.3535 (11)	0.4984 (10)	0.0179 (4)	0.127 (2)
H16D	0.4235	0.3431	0.5213	0.027*	0.127 (2)
H16E	0.2618	0.2923	0.5425	0.027*	0.127 (2)
H16F	0.2565	0.4236	0.5040	0.027*	0.127 (2)
N1	0.18488 (15)	−0.05031 (11)	0.27614 (10)	0.0160 (3)	
N2	−0.08327 (15)	0.12932 (11)	0.21968 (10)	0.0137 (2)	
N3	0.10106 (16)	0.54665 (11)	0.26911 (10)	0.0163 (3)	
N4	0.32667 (15)	0.35576 (11)	0.37691 (10)	0.0146 (2)	
O1	0.16438 (17)	0.14540 (12)	−0.08973 (11)	0.0326 (3)	
O2	0.4842 (2)	0.41629 (15)	−0.06992 (14)	0.0542 (5)	

### Atomic displacement parameters (Å^2^)

**Table:**

	*U*^11^	*U*^22^	*U*^33^	*U*^12^	*U*^13^	*U*^23^
Fe1	0.01141 (10)	0.01167 (10)	0.01369 (10)	−0.00240 (7)	−0.00188 (7)	−0.00282 (8)
Fe2	0.01255 (10)	0.01202 (10)	0.01194 (10)	−0.00203 (8)	−0.00163 (7)	−0.00344 (8)
Ni3	0.01734 (10)	0.01883 (11)	0.01431 (10)	−0.00452 (8)	0.00068 (7)	−0.00536 (8)
S1	0.01209 (16)	0.01827 (18)	0.01966 (18)	−0.00310 (13)	−0.00083 (13)	−0.00635 (15)
S2	0.01514 (17)	0.01348 (17)	0.01239 (16)	−0.00337 (13)	−0.00058 (12)	−0.00372 (13)
S3	0.01420 (17)	0.01595 (18)	0.01823 (18)	−0.00223 (13)	−0.00011 (13)	−0.00679 (14)
S4	0.01799 (17)	0.01352 (17)	0.01314 (16)	−0.00435 (13)	−0.00200 (13)	−0.00111 (13)
C1	0.0172 (8)	0.0192 (8)	0.0312 (9)	0.0001 (6)	0.0009 (6)	−0.0099 (7)
C2	0.0194 (8)	0.0162 (8)	0.0255 (8)	−0.0008 (6)	−0.0006 (6)	−0.0096 (6)
C3	0.0189 (7)	0.0141 (7)	0.0211 (8)	−0.0060 (6)	−0.0020 (6)	−0.0051 (6)
C4	0.0160 (7)	0.0164 (7)	0.0216 (8)	−0.0046 (6)	−0.0034 (6)	−0.0081 (6)
C5	0.0149 (7)	0.0174 (7)	0.0146 (7)	−0.0013 (6)	−0.0042 (5)	−0.0040 (6)
C6	0.0153 (7)	0.0160 (7)	0.0146 (7)	0.0002 (5)	−0.0035 (5)	−0.0034 (6)
C7	0.0265 (8)	0.0152 (8)	0.0243 (8)	−0.0022 (6)	−0.0092 (7)	−0.0009 (6)
C8	0.0144 (7)	0.0192 (8)	0.0160 (7)	−0.0037 (6)	0.0007 (5)	−0.0045 (6)
C9	0.0194 (8)	0.0171 (8)	0.0268 (8)	0.0013 (6)	−0.0065 (6)	−0.0069 (7)
C10	0.0220 (8)	0.0128 (7)	0.0230 (8)	0.0001 (6)	−0.0041 (6)	−0.0059 (6)
C15	0.0357 (10)	0.0173 (8)	0.0159 (7)	−0.0050 (7)	−0.0054 (7)	−0.0006 (6)
C17	0.0215 (8)	0.0216 (8)	0.0208 (8)	0.0032 (6)	−0.0030 (6)	−0.0085 (7)
C18	0.0265 (9)	0.0300 (10)	0.0227 (8)	−0.0067 (7)	0.0054 (7)	−0.0054 (7)
C11A	0.0210 (8)	0.0065 (16)	0.0164 (14)	−0.0054 (9)	−0.0018 (7)	0.0006 (12)
C12A	0.0185 (8)	0.0157 (8)	0.0174 (8)	−0.0044 (6)	−0.0033 (6)	−0.0060 (7)
C13A	0.0210 (9)	0.0168 (9)	0.0174 (8)	−0.0032 (7)	−0.0093 (7)	−0.0012 (7)
C14A	0.0208 (9)	0.0154 (8)	0.0191 (18)	−0.0020 (8)	−0.0099 (9)	−0.0008 (13)
C16A	0.0153 (8)	0.0186 (9)	0.0210 (9)	−0.0038 (7)	0.0010 (6)	−0.0080 (7)
C11B	0.0210 (8)	0.0065 (16)	0.0164 (14)	−0.0054 (9)	−0.0018 (7)	0.0006 (12)
C12B	0.0185 (8)	0.0157 (8)	0.0174 (8)	−0.0044 (6)	−0.0033 (6)	−0.0060 (7)
C13B	0.0210 (9)	0.0168 (9)	0.0174 (8)	−0.0032 (7)	−0.0093 (7)	−0.0012 (7)
C14B	0.0208 (9)	0.0154 (8)	0.0191 (18)	−0.0020 (8)	−0.0099 (9)	−0.0008 (13)
C16B	0.0153 (8)	0.0186 (9)	0.0210 (9)	−0.0038 (7)	0.0010 (6)	−0.0080 (7)
N1	0.0151 (6)	0.0137 (6)	0.0184 (6)	−0.0022 (5)	−0.0025 (5)	−0.0038 (5)
N2	0.0141 (6)	0.0132 (6)	0.0141 (6)	−0.0027 (5)	−0.0017 (5)	−0.0040 (5)
N3	0.0210 (6)	0.0133 (6)	0.0145 (6)	−0.0033 (5)	−0.0028 (5)	−0.0033 (5)
N4	0.0150 (6)	0.0131 (6)	0.0153 (6)	−0.0029 (5)	−0.0017 (5)	−0.0035 (5)
O1	0.0367 (7)	0.0340 (7)	0.0341 (7)	0.0055 (6)	−0.0133 (6)	−0.0223 (6)
O2	0.0591 (11)	0.0505 (10)	0.0465 (10)	−0.0323 (9)	0.0199 (8)	−0.0022 (8)

### Geometric parameters (Å, °)

**Table:**

Fe1—N2	2.2044 (12)	C8—H8C	0.9600
Fe1—N1	2.2408 (13)	C9—C10	1.521 (2)
Fe1—S1	2.3570 (4)	C9—H9A	0.9700
Fe1—S4	2.3734 (4)	C9—H9B	0.9700
Fe1—S2	2.4519 (4)	C10—N3	1.484 (2)
Fe1—Ni3	2.8505 (3)	C10—H10A	0.9700
Fe1—Fe2	3.0945 (3)	C10—H10B	0.9700
Fe2—N4	2.1601 (13)	C15—N3	1.475 (2)
Fe2—S3	2.3122 (4)	C15—H15A	0.9600
Fe2—N3	2.3809 (13)	C15—H15B	0.9600
Fe2—S2	2.3919 (4)	C15—H15C	0.9600
Fe2—S4	2.4511 (4)	C17—O1	1.148 (2)
Ni3—C17	1.7506 (17)	C18—O2	1.138 (2)
Ni3—C18	1.7821 (18)	C11A—N3	1.477 (3)
Ni3—S1	2.3193 (4)	C11A—C12A	1.535 (3)
Ni3—S2	2.3648 (4)	C11A—H11A	0.9700
S1—C1	1.8316 (17)	C11A—H11B	0.9700
S2—C6	1.8361 (15)	C12A—N4	1.474 (2)
S3—C9	1.8240 (17)	C12A—H12A	0.9700
S4—C14A	1.829 (3)	C12A—H12B	0.9700
S4—C14B	1.851 (19)	C13A—N4	1.472 (2)
C1—C2	1.523 (2)	C13A—C14A	1.525 (5)
C1—H1A	0.9700	C13A—H13A	0.9700
C1—H1B	0.9700	C13A—H13B	0.9700
C2—N1	1.481 (2)	C14A—H14A	0.9700
C2—H2A	0.9700	C14A—H14B	0.9700
C2—H2B	0.9700	C16A—N4	1.494 (2)
C3—N1	1.481 (2)	C16A—H16A	0.9600
C3—C4	1.518 (2)	C16A—H16B	0.9600
C3—H3A	0.9700	C16A—H16C	0.9600
C3—H3B	0.9700	C11B—C12B	1.42 (3)
C4—N2	1.4823 (19)	C11B—N3	1.523 (18)
C4—H4A	0.9700	C11B—H11C	0.9700
C4—H4B	0.9700	C11B—H11D	0.9700
C5—N2	1.4851 (19)	C12B—N4	1.420 (12)
C5—C6	1.518 (2)	C12B—H12C	0.9700
C5—H5A	0.9700	C12B—H12D	0.9700
C5—H5B	0.9700	C13B—N4	1.488 (13)
C6—H6A	0.9700	C13B—C14B	1.54 (4)
C6—H6B	0.9700	C13B—H13C	0.9700
C7—N1	1.476 (2)	C13B—H13D	0.9700
C7—H7A	0.9600	C14B—H14C	0.9700
C7—H7B	0.9600	C14B—H14D	0.9700
C7—H7C	0.9600	C16B—N4	1.604 (12)
C8—N2	1.4843 (19)	C16B—H16D	0.9600
C8—H8A	0.9600	C16B—H16E	0.9600
C8—H8B	0.9600	C16B—H16F	0.9600
			
N2—Fe1—N1	80.24 (5)	H9A—C9—H9B	108.1
N2—Fe1—S1	139.76 (3)	N3—C10—C9	111.84 (13)
N1—Fe1—S1	84.02 (3)	N3—C10—H10A	109.2
N2—Fe1—S4	108.57 (3)	C9—C10—H10A	109.2
N1—Fe1—S4	107.46 (4)	N3—C10—H10B	109.2
S1—Fe1—S4	111.447 (15)	C9—C10—H10B	109.2
N2—Fe1—S2	82.61 (3)	H10A—C10—H10B	107.9
N1—Fe1—S2	150.54 (4)	N3—C15—H15A	109.5
S1—Fe1—S2	94.015 (15)	N3—C15—H15B	109.5
S4—Fe1—S2	100.626 (14)	H15A—C15—H15B	109.5
N2—Fe1—Ni3	98.29 (3)	N3—C15—H15C	109.5
N1—Fe1—Ni3	106.94 (3)	H15A—C15—H15C	109.5
S1—Fe1—Ni3	51.843 (11)	H15B—C15—H15C	109.5
S4—Fe1—Ni3	139.189 (13)	O1—C17—Ni3	175.39 (16)
S2—Fe1—Ni3	52.316 (10)	O2—C18—Ni3	175.96 (19)
N2—Fe1—Fe2	99.68 (3)	N3—C11A—C12A	109.62 (19)
N1—Fe1—Fe2	157.78 (3)	N3—C11A—H11A	109.7
S1—Fe1—Fe2	108.341 (13)	C12A—C11A—H11A	109.7
S4—Fe1—Fe2	51.214 (11)	N3—C11A—H11B	109.7
S2—Fe1—Fe2	49.442 (10)	C12A—C11A—H11B	109.7
Ni3—Fe1—Fe2	95.102 (8)	H11A—C11A—H11B	108.2
N4—Fe2—S3	128.04 (4)	N4—C12A—C11A	112.07 (19)
N4—Fe2—N3	77.79 (5)	N4—C12A—H12A	109.2
S3—Fe2—N3	84.35 (3)	C11A—C12A—H12A	109.2
N4—Fe2—S2	115.98 (4)	N4—C12A—H12B	109.2
S3—Fe2—S2	114.298 (15)	C11A—C12A—H12B	109.2
N3—Fe2—S2	96.66 (3)	H12A—C12A—H12B	107.9
N4—Fe2—S4	84.21 (4)	N4—C13A—C14A	113.18 (18)
S3—Fe2—S4	99.308 (15)	N4—C13A—H13A	108.9
N3—Fe2—S4	159.44 (3)	C14A—C13A—H13A	108.9
S2—Fe2—S4	100.131 (14)	N4—C13A—H13B	108.9
N4—Fe2—Fe1	103.49 (3)	C14A—C13A—H13B	108.9
S3—Fe2—Fe1	117.732 (13)	H13A—C13A—H13B	107.8
N3—Fe2—Fe1	145.50 (3)	C13A—C14A—S4	110.2 (3)
S2—Fe2—Fe1	51.154 (10)	C13A—C14A—H14A	109.6
S4—Fe2—Fe1	49.007 (10)	S4—C14A—H14A	109.6
C17—Ni3—C18	115.76 (8)	C13A—C14A—H14B	109.6
C17—Ni3—S1	114.68 (6)	S4—C14A—H14B	109.6
C18—Ni3—S1	107.23 (6)	H14A—C14A—H14B	108.1
C17—Ni3—S2	117.38 (5)	N4—C16A—H16A	109.5
C18—Ni3—S2	102.18 (6)	N4—C16A—H16B	109.5
S1—Ni3—S2	97.362 (15)	N4—C16A—H16C	109.5
C17—Ni3—Fe1	104.28 (6)	C12B—C11B—N3	118 (2)
C18—Ni3—Fe1	139.94 (6)	C12B—C11B—H11C	107.9
S1—Ni3—Fe1	53.046 (11)	N3—C11B—H11C	107.9
S2—Ni3—Fe1	55.140 (11)	C12B—C11B—H11D	107.9
C1—S1—Ni3	109.86 (6)	N3—C11B—H11D	107.9
C1—S1—Fe1	100.08 (5)	H11C—C11B—H11D	107.2
Ni3—S1—Fe1	75.111 (13)	C11B—C12B—N4	104.5 (15)
C6—S2—Ni3	116.05 (5)	C11B—C12B—H12C	110.9
C6—S2—Fe2	102.65 (5)	N4—C12B—H12C	110.9
Ni3—S2—Fe2	134.701 (17)	C11B—C12B—H12D	110.9
C6—S2—Fe1	98.77 (5)	N4—C12B—H12D	110.9
Ni3—S2—Fe1	72.544 (12)	H12C—C12B—H12D	108.9
Fe2—S2—Fe1	79.404 (13)	N4—C13B—C14B	111.5 (14)
C9—S3—Fe2	98.25 (6)	N4—C13B—H13C	109.3
C14A—S4—C14B	9.0 (10)	C14B—C13B—H13C	109.3
C14A—S4—Fe1	108.34 (8)	N4—C13B—H13D	109.3
C14B—S4—Fe1	99.3 (10)	C14B—C13B—H13D	109.3
C14A—S4—Fe2	97.46 (16)	H13C—C13B—H13D	108.0
C14B—S4—Fe2	96.0 (13)	C13B—C14B—S4	116 (2)
Fe1—S4—Fe2	79.779 (13)	C13B—C14B—H14C	108.2
C2—C1—S1	112.85 (11)	S4—C14B—H14C	108.2
C2—C1—H1A	109.0	C13B—C14B—H14D	108.2
S1—C1—H1A	109.0	S4—C14B—H14D	108.2
C2—C1—H1B	109.0	H14C—C14B—H14D	107.3
S1—C1—H1B	109.0	N4—C16B—H16D	109.5
H1A—C1—H1B	107.8	N4—C16B—H16E	109.5
N1—C2—C1	112.68 (13)	H16D—C16B—H16E	109.5
N1—C2—H2A	109.1	N4—C16B—H16F	109.5
C1—C2—H2A	109.1	H16D—C16B—H16F	109.5
N1—C2—H2B	109.1	H16E—C16B—H16F	109.5
C1—C2—H2B	109.1	C7—N1—C3	109.93 (13)
H2A—C2—H2B	107.8	C7—N1—C2	110.37 (13)
N1—C3—C4	110.94 (12)	C3—N1—C2	110.25 (12)
N1—C3—H3A	109.5	C7—N1—Fe1	111.07 (10)
C4—C3—H3A	109.5	C3—N1—Fe1	108.42 (9)
N1—C3—H3B	109.5	C2—N1—Fe1	106.74 (9)
C4—C3—H3B	109.5	C4—N2—C8	110.02 (12)
H3A—C3—H3B	108.0	C4—N2—C5	109.08 (12)
N2—C4—C3	111.19 (12)	C8—N2—C5	110.03 (12)
N2—C4—H4A	109.4	C4—N2—Fe1	107.80 (9)
C3—C4—H4A	109.4	C8—N2—Fe1	105.61 (9)
N2—C4—H4B	109.4	C5—N2—Fe1	114.21 (9)
C3—C4—H4B	109.4	C15—N3—C11A	108.35 (18)
H4A—C4—H4B	108.0	C15—N3—C10	109.56 (13)
N2—C5—C6	112.18 (12)	C11A—N3—C10	110.6 (2)
N2—C5—H5A	109.2	C15—N3—C11B	115.6 (14)
C6—C5—H5A	109.2	C11A—N3—C11B	10.1 (12)
N2—C5—H5B	109.2	C10—N3—C11B	112.4 (14)
C6—C5—H5B	109.2	C15—N3—Fe2	112.91 (10)
H5A—C5—H5B	107.9	C11A—N3—Fe2	108.45 (12)
C5—C6—S2	111.27 (10)	C10—N3—Fe2	107.01 (9)
C5—C6—H6A	109.4	C11B—N3—Fe2	98.7 (11)
S2—C6—H6A	109.4	C12B—N4—C13A	136.8 (5)
C5—C6—H6B	109.4	C12B—N4—C12A	51.1 (5)
S2—C6—H6B	109.4	C13A—N4—C12A	110.12 (13)
H6A—C6—H6B	108.0	C12B—N4—C13B	113.3 (7)
N1—C7—H7A	109.5	C13A—N4—C13B	55.2 (5)
N1—C7—H7B	109.5	C12A—N4—C13B	143.5 (5)
H7A—C7—H7B	109.5	C12B—N4—C16A	60.8 (5)
N1—C7—H7C	109.5	C13A—N4—C16A	109.72 (13)
H7A—C7—H7C	109.5	C12A—N4—C16A	110.66 (13)
H7B—C7—H7C	109.5	C13B—N4—C16A	57.7 (5)
N2—C8—H8A	109.5	C12B—N4—C16B	106.9 (7)
N2—C8—H8B	109.5	C13A—N4—C16B	50.8 (5)
H8A—C8—H8B	109.5	C12A—N4—C16B	61.5 (5)
N2—C8—H8C	109.5	C13B—N4—C16B	103.5 (7)
H8A—C8—H8C	109.5	C16A—N4—C16B	139.9 (4)
H8B—C8—H8C	109.5	C12B—N4—Fe2	111.3 (5)
C10—C9—S3	110.20 (11)	C13A—N4—Fe2	111.61 (10)
C10—C9—H9A	109.6	C12A—N4—Fe2	107.49 (10)
S3—C9—H9A	109.6	C13B—N4—Fe2	109.0 (5)
C10—C9—H9B	109.6	C16A—N4—Fe2	107.19 (10)
S3—C9—H9B	109.6	C16B—N4—Fe2	112.6 (4)
